# *In situ* nanoscale evaluation of pressure-induced changes in structural morphology of phosphonium phosphate ionic liquid at single-asperity contacts†

**DOI:** 10.1039/d1ra08026a

**Published:** 2021-12-22

**Authors:** Zixuan Li, Oscar Morales-Collazo, Robert Chrostowski, Joan F. Brennecke, Filippo Mangolini

**Affiliations:** Texas Materials Institute, The University of Texas at Austin Austin TX 78712 USA filippo.mangolini@austin.utexas.edu; Materials Science and Engineering Program, The University of Texas at Austin Austin TX 78712 USA; McKetta Department of Chemical Engineering, The University of Texas at Austin Austin TX 78712 USA; Walker Department of Mechanical Engineering, The University of Texas at Austin Austin TX 78712 USA

## Abstract

In this work, we perform atomic force microscopy (AFM) experiments to evaluate *in situ* the dependence of the structural morphology of trihexyltetradecylphosphonium bis(2-ethylhexyl) phosphate ([P_6,6,6,14_][DEHP]) ionic liquid (IL) on applied pressure. The experimental results obtained upon sliding a diamond-like-carbon-coated silicon AFM tip on mechanically polished steel at an applied pressure up to 5.5 ± 0.3 GPa indicate a structural transition of confined [P_6,6,6,14_][DEHP] molecules. This pressure-induced morphological change of [P_6,6,6,14_][DEHP] IL leads to the generation of a lubricious, solid-like interfacial layer, whose growth rate increases with applied pressure and temperature. The structural variation of [P_6,6,6,14_][DEHP] IL is proposed to derive from the well-ordered layering of the polar groups of ions separated by the apolar tails. These results not only shed new light on the structural organization of phosphonium-based ILs under elevated pressure, but also provide novel insights into the normal pressure-dependent lubrication mechanisms of ILs in general.

## Abstract 2

In this work, we perform atomic force microscopy (AFM) experiments to evaluate *in situ* the dependence of the structural morphology of trihexyltetradecylphosphonium bis(2-ethylhexyl) phosphate ([P_6,6,6,14_][DEHP]) ionic liquid (IL) on applied pressure.

## Graphical abstract

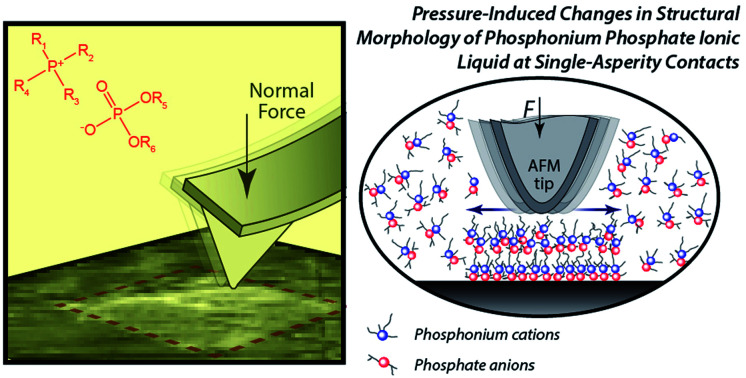

## 1. Introduction

Ionic liquids (ILs) consist of organic cations and weakly coordinating anions. The low melting temperature of ILs (<100 °C) derives from the combined effect of the size difference between the ions, the geometric asymmetry of ions, and charge delocalization of at least one of the two ions, which weakens the electrostatic interactions between ions and increases their configurational entropy.^1,2^ The unique physico-chemical properties (*e.g.*, negligible vapor pressure, wide electrochemical window, high thermal stability, low flammability) and the high “tunability” of ILs, which derives from the virtually unlimited number of permutations of cations and anions (10^18^ ILs available^3^), have paved the way towards tailoring the structures and functionalities of ILs to achieve task-specific properties.^4^ Therefore, ILs are usually referred to as “designer solvents”, emerging as candidate materials for a variety of applications, including solvents in catalysis,^5^ reaction media,^6^ electrolytes in energy storage devices,^7^ active pharmaceutical ingredients,^8,9^ and lubricating fluids.^10,11^ In all these applications, the functional behaviors (*e.g.*, catalysts' selectivity, charge storage of supercapacitors) depend on the response of ILs to external stimuli, such as electric field, pressure, and temperature. Because of this, the dependence of the properties and structures of ILs on externally controlled parameters has been the subject of extensive research.^12–30^ In the case of the dependence of the IL structure on applied pressure, several studies employed surface force apparatuses (SFA) and colloidal atomic force microscopy (AFM) to evaluate the structure of IL/solid interfaces upon nanoconfinement.^1,2,31–34^ SFA and colloidal AFM studies, in which the maximum uniaxial compressive normal stress during nanoconfinement studies is typically in the MPa range, showed the presence of damped oscillations in the measured force profiles as the distance between the two confining surfaces is reduced, thus indicating the formation of an ordered, layered interfacial structure that is difficult to be squeezed out.^1,35–37^ The dynamic behaviors of nanoconfined ILs have also been investigated by SFA and colloidal AFM as the viscosity and phase response of these fluids in confined geometry strongly affect the lubricating property of these fluids.^1,11,36,38,39^ In the last decade, an increasing number of studies also evaluated the structures and physico-chemical properties of imidazolium- and pyrrolidinium-based ILs as a function of hydrostatic pressure.^40–57^ Zhao *et al.* employed molecular dynamics (MD) simulations to investigate the effect of pressure on the interionic interactions of 1-butyl-3-methylimidazolium hexafluorophosphate ([C_4_mim][PF_6_]) IL and provided evidence for changes in the conformation in the alkyl chains of cations at high pressure (0.6 GPa).^41^ Saouane *et al.* evaluated the solid-state polymorphism of the same IL using single-crystal X-ray diffraction, Raman spectroscopy, and optical microscopy.^57^ The experimental results indicated the existence of three polymorphs, which was proposed to originate from the conformational flexibility of [C_4_mim] cations together with the rotational disorder of [PF_6_] anions. While other studies provided further evidence for pressure-dependent structural transitions in imidazolium-based ILs as a consequence of conformational variations in cationic tails,^40,53,56,58^ MD simulations by Russina *et al.* not only confirmed the pressure-induced change of the dihedral angles along cationic alkyl chains, but also demonstrated that polar moieties interacting through coulombic interactions are less affected by the applied pressure.^52^ More recently, Sharma *et al.* employed MD simulations to identify structural variations in 1-alkyl-1-methylpyrrolidinium bis(trifluoromethylsulfonyl)amide ([Pyrr_1,*n*_][NTf_2_], where *n* = 8 or 10) and demonstrated the susceptibility of both apolar and polar groups to changes in applied pressure, while also showing that [NTf_2_] anions could undergo conformational changes.^42^

Among the ILs that have been studied, phosphonium-based ILs are particularly attractive owing to their combination of high thermal stability, good solubility of carbon dioxide and organic/inorganic solutes,^59–61^ and good lubricating properties.^62–64^ While a few works reported the evolution of densities and thermodynamic properties for phosphonium-based ILs as a function of applied pressure,^65,66^ a limited number of studies have been reported about pressure-induced structural changes in these ILs. Sharma *et al.* used MD simulations to investigate variations in structural morphology of trihexyl(tetradecyl)phosphonium bromide ([P_6,6,6,14_][Br]) and trihexyl(tetradecyl)phosphonium dicyanamide ([P_6,6,6,14_][DCA]) ILs.^67^ Upon increasing the applied pressure, a crystalline order forms due to increased polar–polar and apolar–apolar correlations within the ILs. Moreover, the simulations clearly indicated the formation of a well-ordered, solid-like layering of the polar moieties separated by the apolar tails of the cations at hydrostatic pressures above 0.1 GPa for [P_6,6,6,14_][DCA] and 0.2 GPa for [P_6,6,6,14_][Br].

**Fig. 1:**
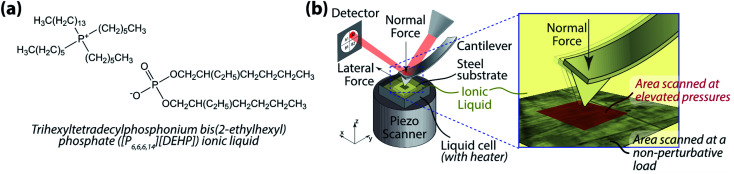
(a) [P_6,6,6,14_][DEHP] IL; and (b) schematic diagram of the *in situ* atomic force microscopy (AFM) approach used to evaluate the pressure-dependent structural morphology of [P_6,6,6,14_][DEHP] IL.

While the study by Sharma *et al.*^67^ provided fundamental insights into the intrinsic structural transitions of phosphonium-based ILs under hydrostatic pressure using MD simulations, no experimental study has yet to be reported to corroborate the results. Additionally, remarkably little has been discussed about the functional aspects of this pressure-induced behavior of ILs in general. Here, we use atomic force microscopy (AFM) to evaluate *in situ* the evolution of the structural morphology of trihexyltetradecylphosphonium bis(2-ethylhexyl) phosphate ([P_6,6,6,14_][DEHP]) IL (Fig. 1a) under accurately controlled normal pressure, and explore the relation between the IL's structural change and its frictional behavior.

## 2. Materials and methods

AFM experiments were carried out using a commercial MFP-3D Origin+ AFM (Oxford Instruments, Asylum Research, USA). Polished, air-oxidized 52 100 steel disks (McMaster Carr, USA; root-mean-square roughness over a 5 × 5 μm^2^ area: 2.1 ± 0.4 nm) were used as the substrates. During the experiments, a few drops (∼0.1 g) of [P_6,6,6,14_][DEHP] IL were placed on the substrate to ensure complete immersion of the AFM cantilever in the IL once engaged. The synthetic procedure for [P_6,6,6,14_][DEHP] IL is reported in ref. 68. The water content of this IL was 17 139 ± 1549 ppm at room temperature (22 ± 1 °C) as determined by a Brinkman 831 Karl Fischer coulometer before tests. The normal spring constant of the AFM cantilever was calibrated using the Sader's method.^69,70^ To compute the applied normal pressure, the tip shape was characterized before and after each experiment using the blind tip reconstruction method^71^ employing an ultra-nanocrystalline diamond surface (Aqua 25, Advanced Diamond Technologies, USA) as the reference substrate. To avoid any significant changes in tip shape during the experiments, diamond-like carbon (DLC)-coated silicon tips (NSC14-Al-BS, Mikromasch, USA. Spring constant ∼5 N m^−1^) were employed. To evaluate changes in structural morphology of [P_6,6,6,14_][DEHP] IL, *in situ* AFM tests were performed by scanning a 2 × 2 μm^2^ area at a scan speed of 78 μm s^−1^. Pressure- and temperature-dependent experiments were performed by varying the applied normal pressure between 2.7 ± 0.3 GPa and 7.3 ± 0.4 GPa (computed from Hertz contact mechanics, assuming the Young's moduli of steel and DLC to be 201 GPa and 150 GPa, respectively) and the temperature between room temperature (22 ± 1 °C) and 111 ± 1 °C. Structural changes induced by the pressure applied by the AFM tip during the sliding process were visualized by periodically acquiring zoomed-out topographical and friction force images (5 × 5 μm^2^) at a non-perturbative load (Fig. 1b).

## 3. Results and discussion

**Fig. 2:**
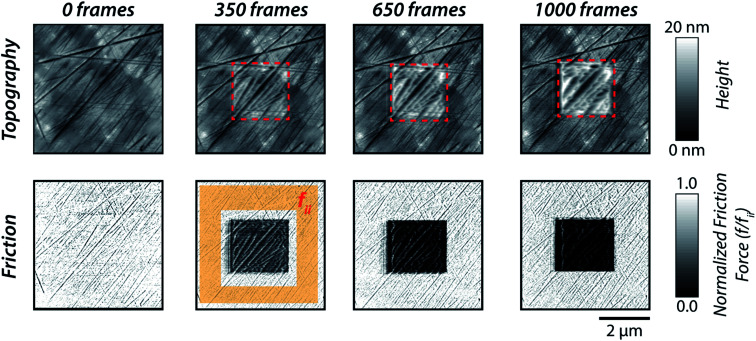
Topographic (top row) and normalized friction force (bottom row) AFM images (5 × 5 μm^2^) of an air-oxidized steel surface obtained using a DLC-coated silicon AFM tip immersed in [P_6,6,6,14_][DEHP] IL at 111 ± 1 °C. The topographic images were collected at a non-perturbative load. The number of 2 × 2 μm^2^ frames, which were previously collected in the central part of the image at an applied pressure of 5.5 ± 0.3 GPa (highlighted with a red, dashed box), is reported above each column of images. The friction force maps were normalized by the average friction force (*f*_ii_) in the region scanned by a non-perturbative load.

**Fig. 3:**
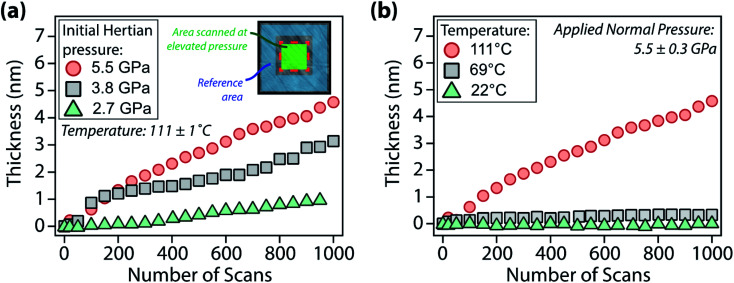
Thickness of the surface layer formed by [P_6,6,6,14_][DEHP] IL on air-oxidized steel as a function of the number of scans for the case of: (a) experiments performed at different applied normal pressures (temperature: 111 ± 1 °C); and (b) experiments carried out at different temperatures (applied normal pressure: 5.5 ± 0.3 GPa). The layer thickness was obtained by computing the height difference between the mean plane of the area scanned at high pressure (green shaded area in the AFM topographic image in the inset in (a)) and the mean plane of the reference area (blue shaded area in the AFM topographic image in the inset in (a)).

The AFM experiments performed at 111 ± 1 °C indicated a variation in nanoscale structural morphology of [P_6,6,6,14_][DEHP] IL upon scanning at an applied average normal pressure of 5.5 ± 0.3 GPa. Fig. 2 displays typical zoomed-out topography images (5 × 5 μm^2^) of air-oxidized steel obtained at a non-perturbative load together with the corresponding normalized friction force maps. These height maps were acquired after the AFM tip scanned the central area (2 × 2 μm^2^) at 5.5 ± 0.3 GPa for different numbers of frames. The topographic images reveal the progressive formation of an interfacial layer at randomly located nucleation sites, whose thickness subsequently increased over the course of 1000 scan cycles (Fig. 3a). Correspondingly, an increase in contrast in the friction force maps was detected (Fig. 2), which indicated a reduction in friction force with pressure-induced changes in structural morphology of [P_6,6,6,14_][DEHP] IL occurring upon scanning at 5.5 ± 0.3 GPa and 111 ± 1 °C.

The sample topography after removing the supernatant [P_6,6,6,14_][DEHP] IL and sonicating the substrate with organic solvents (methanol and isopropanol) revealed the complete removal of the interfacial layer and the absence of any mechanical wear of the underlying air-oxidized steel substrate (see Fig. S1 in the ESI†). The absence of any contrast in the friction force map acquired on the sample after its sonication in organic solvents (see Fig. S2 in the ESI†) is also indicative of no changes in surface chemistry in the region scanned at elevated pressure, thus corroborating the lack of any stress-assisted chemical reaction of the IL on the steel surface. The formation of a non-surface-bound reaction layer, as observed in our AFM experiments, is in contrast to macroscale sliding experiments with the same IL,^62–64,72,73^ which indicated the stress-assisted, thermally-activated chemical reaction of [P_6,6,6,14_][DEHP] on steel or cast iron surfaces to generate a mechanically-stable iron phosphate film (as thick as 120–180 nm (ref. 64)). The difference between our work and previously published studies can be ascribed to the completely different contact conditions employed in the experiments: while the low sliding speed of the AFM tip resulted in a negligible temperature rise (≪1 °C) at the contact, the high sliding speeds of multi-asperity, macroscale sliding contacts could drastically increase the contact temperature (as high as 140 °C),^68^ thus accelerating any surface mechano-chemical reaction of the [P_6,6,6,14_][DEHP] IL. In light of this, the variations in topographic AFM images observed in the present study can be attributed to a pressure-induced change in structural morphology of the [P_6,6,6,14_][DEHP] molecules. The resulting solid-like interfacial layer is proposed to derive from the well-ordered layering of polar groups of [P_6,6,6,14_][DEHP] ions separated by apolar tails, as suggested by MD simulations performed on phosphonium-based ILs.^67^ This finding also indicates that the effect of the nanoconfinement of ILs results in the formation of a solid-like structure that remains (at least in part) on the substrate surface after the release of the applied force.

It is also critical to highlight that, while the absence of any stress-assisted surface reaction in the experiments presented herein is in agreement with our previous work,^68^ the formation of the surface layer shown in Fig. 2 upon sliding at 5.5 ± 0.3 GPa contrasts with the results obtained from AFM experiments carried out at a higher applied pressure (7.3 ± 0.4 GPa) in the same previous study. In the latter case, the topographic AFM images indicate progressive material removal (*i.e.*, wear) from the air-oxidized steel upon sliding, thus suggesting that any interfacial layer formed as a result of pressure-induced changes in structural morphology of [P_6,6,6,14_][DEHP] IL is not mechanically stable at 7.3 ± 0.4 GPa. Despite this difference in the evolution of the surface topography upon sliding at elevated pressures, a decrease in nanoscale friction was measured in the area slid at high loads in both scenarios (5.5 ± 0.3 GPa and 7.3 ± 0.4 GPa (ref. 68)), which indicates that different mechanisms underpin the measured friction reduction: in the present study, the decrease in friction in the area scanned at 5.5 ± 0.3 GPa can be ascribed to the pressure-induced variation in structure of [P_6,6,6,14_][DEHP] IL, whereas in our previous work the friction reduction was shown to be due to the adsorption of phosphate ions on the mechanically smoothened metallic iron surface that leads to the formation of a densely-packed, lubricious boundary film.^68^ This finding sheds new light on the dependence of the lubrication mechanism of phosphonium phosphate ILs on applied normal pressure, as it demonstrates that pressure-induced changes in structural morphology of phosphonium phosphate ILs can control the nanoscale friction response through the formation of a well-defined interfacial layer that is mechanically stable up to a critical applied normal pressure, above which wear occurs and the surface adsorption of phosphate ions on freshly-exposed metallic iron surfaces dictates the friction response at the nanoscale.

To evaluate the pressure- and temperature-dependence of the structural changes of [P_6,6,6,14_][DEHP], AFM experiments were performed at different applied normal forces and temperatures. Fig. 3 displays the evolution of the thickness of the interfacial layer as a function of the number of scans. Increasing the applied normal pressure from 2.7 ± 0.3 GPa to 5.5 ± 0.3 GPa at a constant temperature (111 ± 1 °C) progressively increases the thickness and growth rate of the interfacial layer with the same number of scans (Fig. 3a and S3 in the ESI†). Additionally, while at lower temperatures (22 ± 1 °C and 69 ± 1 °C) the AFM topographic images indicated the generation of an extremely thin interfacial layer (thickness <1 nm), increasing the temperature to 111 ± 1 °C significantly enhances the layer formation (Fig. 3b). The increase in growth rate of the solid-like interfacial layer upon increasing the IL temperature can be attributed to the higher mobility (or self-diffusivity) of the ions in [P_6,6,6,14_][DEHP] at elevated temperatures (the self-diffusivity *D*_i_ of a species i with radius *r*_i_ is inversely proportional to the viscosity of the medium *η* according to Stokes–Einstein relation: 
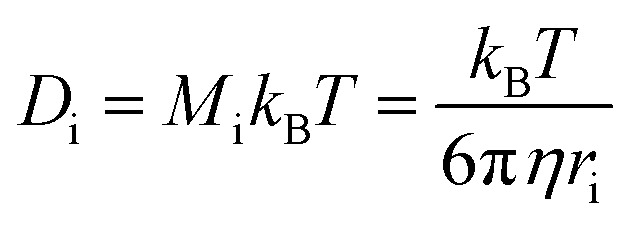
, where *M*_i_ is the mobility of species i, *k*_B_ is the Boltzmann constant and *T* is the temperature. The variation of the viscosity of [P_6,6,6,14_][DEHP] with temperature is shown in Fig. S4 in the ESI†), which makes the ions more responsive to the mechanical action of the AFM probe. An additional factor that might affect the growth rate of the solid-like interfacial layer is related to the water content in [P_6,6,6,14_][DEHP] (*i.e.*, 17 139 ± 1549 ppm at 22 ± 1 °C). Thermogravimetric measurements indicated that a mass loss occurs between 100 °C and 110 °C (see Fig. S5 in the ESI†), which is likely due to water desorption from [P_6,6,6,14_][DEHP]. Thus, while the significant amount of water present in [P_6,6,6,14_][DEHP] at 22 ± 1 °C and 69 ± 1 °C can hydrogen-bond with the IL ions ([DEHP] anions in [P_6,6,6,14_][DEHP], in particular), affect the ionic arrangement,^74^ and interfere with the formation of a solid-like, ordered structure by screening ion–ion interactions, in the case of the AFM experiments performed at 111 ± 1 °C the reduced water content in [P_6,6,6,14_][DEHP] allows for the well-ordered layering of polar groups of [P_6,6,6,14_][DEHP] ions separated by apolar tails, thus resulting in a much larger thickness of the interfacial layer.

## 4. Conclusions

In summary, we carried out *in situ* AFM experiments to evaluate the pressure-induced evolution of the structural morphology of [P_6,6,6,14_][DEHP] IL. The AFM results obtained upon sliding a DLC-coated silicon AFM tip on mechanically polished steel at an applied pressure up to 5.5 ± 0.3 GPa indicate the pressure-induced generation of a solid-like, lubricious interfacial layer, whose thickness and growth rate increase with both applied normal pressure and temperature. The interfacial structure created by [P_6,6,6,14_][DEHP], which is proposed to be due to the layering of polar groups of IL ions separated by apolar tails,^67^ is stable upon releasing the applied pressure, but fully removed from the substrate upon removing the supernatant IL and washing the sample with organic solvents. Notably, the pressure-induced change in structural morphology of [P_6,6,6,14_][DEHP] decreases nanoscale friction, while preventing mechanical damage (wear) of the underlying steel substrate. These findings do not only complement and corroborate previously published MD simulations that indicate the occurrence of pressure-dependent polymorphic phase transformations in ILs,^67^ but also provide experimental evidence for a potentially universal mechanism that underpins the nanoscale lubrication mechanism of ILs in general. Further experimental and modeling work is needed to evaluate pressure-induced phase transformations in other classes of ILs and their dependence on the molecular structure of ILs, contaminants in ILs (*e.g.*, water and halides), and externally-controlled parameters (*e.g.*, temperature, applied bias voltage).

## 5 Associated content

Topographic AFM images of an air-oxidized steel acquired with the AFM tip immersed in [P_6,6,6,14_][DEHP] IL (the topographic images were obtained before and after scanning the central part of the frame at an applied pressure of 5.5 ± 0.3 GPa). Topographic image collected in the same sample area at the end of the experiment performed at 5.5 ± 0.3 GPa and after sonicating the sample in methanol and isopropanol. Pressure-dependence of the volumetric growth rate of the interfacial layer formed by [P_6,6,6,14_][DEHP] at 111 ± 1 °C. Dependence of the dynamic viscosity of [P_6,6,6,14_][DEHP] on temperature. Thermogravimetric analysis of [P_6,6,6,14_][DEHP] IL.

## Conflicts of interest

The authors declare no competing financial interests.

## Supplementary Material

RA-012-D1RA08026A-s001

## References

[cit1] Espinosa-Marzal R. M. ; Han M.; Arcifa A.; Spencer N. D. and Rossi A. Ionic Liquids at Interfaces and Their Tribological Behavior, in Reference Module in Chemistry, Molecular Sciences and Chemical Engineering, 2017

[cit2] Perkin S. (2012). Ionic liquids in confined geometries. Phys. Chem. Chem. Phys..

[cit3] Niedermeyer H., Hallett J. P., Villar-Garcia I. J., Hunt P. A., Welton T. (2012). Mixtures of ionic liquids. Chem. Soc. Rev..

[cit4] Lei Z., Chen B., Koo Y. M., MacFarlane D. R. (2017). Introduction: Ionic Liquids. Chem. Rev..

[cit5] Steinrück H.-P., Wasserscheid P. (2014). Ionic Liquids in Catalysis. Catal. Lett..

[cit6] Earle M. J., Seddon K. R. (2000). Ionic liquids. Green solvents for the future. Pure Appl. Chem..

[cit7] MacFarlane D. R., Tachikawa N., Forsyth M., Pringle J. M., Howlett P. C., Elliott G. D., Davis J. H., Watanabe M., Simon P., Angell C. A. (2014). Energy applications of ionic liquids. Energy Environ. Sci..

[cit8] Dean P. M., Turanjanin J., Yoshizawa-Fujita M., MacFarlane D. R., Scott J. L. (2009). Exploring an Anti-Crystal Engineering Approach to the Preparation of Pharmaceutically Active Ionic Liquids. Cryst. Growth Des..

[cit9] Egorova K. S., Gordeev E. G., Ananikov V. P. (2017). Biological Activity of Ionic Liquids and Their Application in Pharmaceutics and Medicine. Chem. Rev..

[cit10] Zhou Y., Qu J. (2017). Ionic Liquids as Lubricant Additives: A Review. ACS Appl. Mater. Interfaces.

[cit11] Li Z., Mangolini F. (2021). Recent Advances in Nanotribology of Ionic Liquids. Exp. Mech..

[cit12] Wishart J. F., Castner E. W. (2007). The Physical Chemistry of Ionic Liquids. J. Phys. Chem. B.

[cit13] Triolo A., Russina O., Fazio B., Appetecchi G. B., Carewska M., Passerini S. (2009). Nanoscale Organization In Piperidinium-Based Room Temperature Ionic Liquids. J. Chem. Phys..

[cit14] Hardacre C., Holbrey J. D., Nieuwenhuyzen M., Youngs T. G. A. (2007). Structure and Solvation in Ionic Liquids. Acc. Chem. Res..

[cit15] Gontrani L., Russina O., Celso F. L., Caminiti R., Annat G., Triolo A. (2009). Liquid Structure of Trihexyltetradecylphosphonium Chloride at Ambient Temperature: An X-Ray Scattering and Simulation Study. J. Phys. Chem. B.

[cit16] Gomes M., Lopes J. N. C., Pádua A. A. H. (2009). Thermodynamics and Micro Heterogeneity of Ionic Liquids. Top. Curr. Chem..

[cit17] Castiglione F., Moreno M., Raos G., Famulari A., Mele A., Appetecchi G. B., Passerini S. (2009). Structural Organization and Transport Properties of Novel Pyrrolidinium-Based Ionic Liquids with Perfluoroalkyl Sulfonylimide Anions. J. Phys. Chem. B.

[cit18] Bradley A. E., Hardacre C., Holbrey J. D., Johnston S., McMath S. E. J., Nieuwenhuyzen M. (2002). Small-Angle X-Ray Scattering Studies of Liquid Crystalline 1-Alkyl-3-Methylimidazolium Salts. Chem. Mater..

[cit19] Atkin R., Warr G. G. (2008). The Smallest Amphiphiles: Nanostructure in Protic Room-Temperature Ionic Liquids with Short Alkyl Groups. J. Phys. Chem. B.

[cit20] Urahata S. M., Ribeiro M. C. (2004). Structure of Ionic Liquids of 1-Alkyl-3-Methylimidazolium Cations: A Systematic Computer Simulation Study. J. Chem. Phys..

[cit21] Siqueira L. J. A., Ribeiro M. C. C. (2011). Charge Ordering and Intermediate Range Order in Ammonium Ionic Liquids. J. Chem. Phys..

[cit22] Shimizu K., Bernardes C. E. S., Canongia Lopes J. N. (2014). Structure and Aggregation in the 1-Alkyl-3-Methylimidazolium Bis(trifluoromethylsulfonyl)imide Ionic Liquid Homologous Series. J. Phys. Chem. B.

[cit23] Kashyap H. K., Santos C. S., Murthy N. S., Hettige J. J., Kerr K., Ramati S., Gwon J., Gohdo M., Lall-Ramnarine S. I., Wishart J. F. (2013). Structure of 1-Alkyl-1-Methylpyrrolidinium Bis(trifluoromethylsulfonyl)amide Ionic Liquids with Linear, Branched, and Cyclic Alkyl Groups. J. Phys. Chem. B.

[cit24] Del Popolo M. G., Voth G. A. (2004). On the Structure and Dynamics of Ionic Liquids. J. Phys. Chem. B.

[cit25] Bhargava B., Klein M., Balasubramanian S. (2008). Structural Correlations and Charge Ordering in a Room-Temperature Ionic Liquid. ChemPhysChem.

[cit26] Annapureddy H. V. R., Kashyap H. K., De Biase P. M., Margulis C. J. (2010). What Is the Origin of the Prepeak in the X-Ray Scattering of Imidazolium-Based Room-Temperature Ionic Liquids?. J. Phys. Chem. B.

[cit27] Russina O. ; Fazio B.; Di Marco G.; Caminiti R.; Triolo A.; Caminiti R. and Gontrani L., The Structure of Ionic Liquids, 2014, p. 39

[cit28] Lauw Y., Horne M. D., Rodopoulos T., Lockett V., Akgun B., Hamilton W. A., Nelson A. R. J. (2012). Structure of [C4mpyr][NTf2] Room-Temperature Ionic Liquid at Charged Gold Interfaces. Langmuir.

[cit29] Di Lecce S., Kornyshev A. A., Urbakh M., Bresme F. (2020). Electrotunable Lubrication with Ionic Liquids: the Effects of Cation Chain Length and Substrate Polarity. ACS Appl. Mater. Interfaces.

[cit30] Bedrov D., Vatamanu J., Hu Z. (2015). Ionic liquids at charged surfaces: Insight from molecular simulations. J. Non-Cryst. Solids.

[cit31] Perez-Martinez C. S., Perkin S. (2019). Interfacial Structure and Boundary Lubrication of a Dicationic Ionic Liquid. Langmuir.

[cit32] Gebbie M. A., Smith A. M., Dobbs H. A., Lee A. A., Warr G. G., Banquy X., Valtiner M., Rutland M. W., Israelachvili J. N., Perkin S., Atkin R. (2016). Long range electrostatic forces in ionic liquids. Chem. Commun..

[cit33] Nalam P. C., Sheehan A., Han M., Espinosa-Marzal R. M. (2020). Effects of Nanoscale Roughness on the Lubricious Behavior of an Ionic Liquid. Adv. Mater. Interfaces.

[cit34] Han M., Espinosa-Marzal R. M. (2019). Influence of Water on Structure, Dynamics, and Electrostatics of Hydrophilic and Hydrophobic Ionic Liquids in Charged and Hydrophilic Confinement between Mica Surfaces. ACS Appl. Mater. Interfaces.

[cit35] Elbourne A., McDonald S., Voichovsky K., Endres F., Warr G. G., Atkin R. (2015). Nanostructure of the Ionic Liquid-Graphite Stern Layer. ACS Nano.

[cit36] Lhermerout R., Diederichs C., Perkin S. (2018). Are Ionic Liquids Good Boundary Lubricants? A Molecular Perspective. Lubricants.

[cit37] Perkin S., Albrecht T., Klein J. (2010). Layering and shear properties of an ionic liquid, 1-ethyl-3-methylimidazolium ethylsulfate, confined to nano-films between mica surfaces. Phys. Chem. Chem. Phys..

[cit38] Somers A., Howlett P., MacFarlane D., Forsyth M. (2013). A Review of Ionic Liquid Lubricants. Lubricants.

[cit39] Lainé A., Niguès A., Bocquet L., Siria A. (2020). Nanotribology of Ionic Liquids: Transition to Yielding Response in Nanometric Confinement with Metallic Surfaces. Phys. Rev. X.

[cit40] Yoshimura Y., Shigemi M., Takaku M., Yamamura M., Takekiyo T., Abe H., Hamaya N., Wakabayashi D., Nishida K., Funamori N. (2015). Stability of the Liquid State of Imidazolium-Based Ionic Liquids under High Pressure at Room Temperature. J. Phys. Chem. B.

[cit41] Zhao Y., Liu X., Lu X., Zhang S., Wang J., Wang H., Gurau G., Rogers R. D., Su L., Li H. (2012). The behavior of ionic liquids under high pressure: a molecular dynamics simulation. J. Phys. Chem. B.

[cit42] Sharma S., Gupta A., Kashyap H. K. (2016). How the Structure of Pyrrolidinium Ionic Liquids Is Susceptible to High Pressure. J. Phys. Chem. B.

[cit43] Chang H.-C., Chang C.-Y., Su J.-C., Chu W.-C., Jiang J.-C., Lin S. H. (2006). Conformations of 1-Butyl-3-methylimidazolium Chloride Probed by High Pressure Raman Spectroscopy. Int. J. Mol. Sci..

[cit44] Su L., Zhu X., Wang Z., Cheng X., Wang Y., Yuan C., Chen Z., Ma C., Li F., Zhou Q., Cui Q. (2012). In situ observation of multiple phase transitions in low-melting ionic liquid [BMIM][BF4] under high pressure up to 30 GPa. J. Phys. Chem. B.

[cit45] Yoshimura Y., Abe H., Takekiyo T., Shigemi M., Hamaya N., Wada R., Kato M. (2013). Superpressing of a room temperature ionic liquid, 1-ethyl-3-methylimidazolium tetrafluoroborate. J. Phys. Chem. B.

[cit46] Li H., Su L., Zhu X., Cheng X., Yang K., Yang G. (2014). In situ crystallization of ionic liquid [Emim][PF6] from methanol solution under high pressure. J. Phys. Chem. B.

[cit47] Dymek C. J., Grossie D. A., Fratini A. V., Wade Adams W. (1989). Evidence for the presence of hydrogen-bonded ion-ion interactions in the molten salt precursor, 1-methyl-3-ethylimidazolium chloride. J. Mol. Struct..

[cit48] Shah J. K., Maginn E. J. (2010). Molecular Dynamics Investigation of Biomimetic Ionic Liquids. Fluid Phase Equilib..

[cit49] Takekiyo T., Hatano N., Imai Y., Abe H., Yoshimura Y. (2011). Pressure-induced Phase Transition of 1-Butyl-3-Methylimidazolium Hexafluorophosphate [bmim][PF6]. High Pressure Res..

[cit50] Imai Y., Takekiyo T., Abe H., Yoshimura Y. (2011). Pressure- and Temperature-induced Raman Spectral Changes of 1-Butyl-3-Methylimidazolium Tetrafluoroborate. High Pressure Res..

[cit51] Su L., Li M., Zhu X., Wang Z., Chen Z., Li F., Zhou Q., Hong S. (2010). In Situ Crystallization of Low-Melting Ionic Liquid [BMIM][PF6] under High Pressure up to 2 GPa. J. Phys. Chem. B.

[cit52] Russina O., Lo Celso F., Triolo A. (2015). Pressure-responsive mesoscopic structures in room temperature ionic liquids. Phys. Chem. Chem. Phys..

[cit53] Yoshimura Y., Takekiyo T., Imai Y., Abe H. (2012). Pressure-Induced Spectral Changes of Room-Temperature Ionic Liquid, N,N-Diethyl-N-Methyl-N-(2-Methoxyethyl)ammonium Bis(trifluoromethylsulfonyl)imide, [DEME][TFSI]. J. Phys. Chem. C.

[cit54] Shimizu K., Bernardes C. E. S., Triolo A., Canongia Lopes J. N. (2013). Nano-segregation in Ionic Liquids: Scorpions and Vanishing Chains. Phys. Chem. Chem. Phys..

[cit55] Faria L. F. O., Ribeiro M. C. C. (2015). Phase Transitions of Triflate-Based Ionic Liquids under High Pressure. J. Phys. Chem. B.

[cit56] Abe H., Takekiyo T., Hatano N., Shigemi M., Hamaya N., Yoshimura Y. (2014). Pressure-Induced Frustration-Frustration Process in 1-Butyl-3-Methylimidazolium Hexafluorophosphate, a Room-Temperature Ionic Liquid. J. Phys. Chem. B.

[cit57] Saouane S., Norman S. E., Hardacre C., Fabbiani F. P. A. (2013). Pinning down the solid-state polymorphism of the ionic liquid [bmim][PF6]. Chem. Sci..

[cit58] Zhu X., Li H., Wang Z., Yuan C., Zhu P., Su L., Yang K., Wu J., Yang G., Li X. (2017). Pressure-induced ionic liquid crystal in 1-dodecyl-3-methylimidazolium tetrafluoroborate. RSC Adv..

[cit59] Fraser K. J., Izgorodina E. I., Forsyth M., Scott J. L., MacFarlane D. R. (2007). Liquids intermediate between "molecular" and "ionic" liquids: liquid ion pairs?. Chem. Commun..

[cit60] Carvalho P. J., Álvarez V. H., Marrucho I. M., Aznar M., Coutinho J. A. P. (2010). High carbon dioxide solubilities in trihexyltetradecylphosphonium-based ionic liquids. J. Supercrit. Fluids.

[cit61] Pison L., Canongia Lopes J. N., Rebelo L. P., Padua A. A., Costa Gomes M. F. (2008). Interactions of fluorinated gases with ionic liquids: solubility of CF4, C2F6, and C3F8 in trihexyltetradecylphosphonium bis(trifluoromethylsulfonyl)amide. J. Phys. Chem. B.

[cit62] Zhou Y., Dyck J., Graham T. W., Luo H., Leonard D. N., Qu J. (2014). Ionic liquids composed of phosphonium cations and organophosphate, carboxylate, and sulfonate anions as lubricant antiwear additives. Langmuir.

[cit63] Barnhill W. C., Qu J., Luo H., Meyer 3rd H. M., Ma C., Chi M., Papke B. L. (2014). Phosphonium-organophosphate ionic liquids as lubricant additives: effects of cation structure on physicochemical and tribological characteristics. ACS Appl. Mater. Interfaces.

[cit64] Qu J., Bansal D. G., Yu B., Howe J. Y., Luo H., Dai S., Li H., Blau P. J., Bunting B. G., Mordukhovich G., Smolenski D. J. (2012). Antiwear Performance and Mechanism of an Oil-Miscible Ionic Liquid as a Lubricant Additive. ACS Appl. Mater. Interfaces.

[cit65] Tomé L. I. N., Gardas R. L., Carvalho P. J., Pastoriza-Gallego M. J., Piñeiro M. M., Coutinho J. o. A. P. (2011). Measurements and Correlation of High-Pressure Densities of Phosphonium Based Ionic Liquids. J. Chem. Eng. Data.

[cit66] Esperança J. M. S. S., Guedes H. J. R., Blesic M., Rebelo L. P. N. (2006). Densities and Derived Thermodynamic Properties of Ionic Liquids. 3. Phosphonium-Based Ionic Liquids over an Extended Pressure Range. J. Chem. Eng. Data.

[cit67] Sharma S., Gupta A., Dhabal D., Kashyap H. K. (2016). Pressure-dependent morphology of trihexyl(tetradecyl)phosphonium ionic liquids: A molecular dynamics study. J. Chem. Phys..

[cit68] Li Z., Dolocan A., Morales-Collazo O., Sadowski J. T., Celio H., Chrostowski R., Brennecke J. F., Mangolini F. (2020). Lubrication Mechanism of Phosphonium Phosphate Ionic Liquid in Nanoscale Single-Asperity Sliding Contacts. Adv. Mater. Interfaces.

[cit69] Sader J. E., Sanelli J. A., Adamson B. D., Monty J. P., Wei X., Crawford S. A., Friend J. R., Marusic I., Mulvaney P., Bieske E. J. (2012). Spring constant calibration of atomic force microscope cantilevers of arbitrary shape. Rev. Sci. Instrum..

[cit70] Sader J. E., Chon J. W. M., Mulvaney P. (1999). Calibration of rectangular atomic force microscope cantilevers. Rev. Sci. Instrum..

[cit71] Liu J., Notbohm J. K., Carpick R. W., Turner K. T. (2010). Method for Characterizing Nanoscale Wear of Atomic Force Microscope Tips. ACS Nano.

[cit72] Guo W., Zhou Y., Sang X., Leonard D. N., Qu J., Poplawsky J. D. (2017). Atom Probe Tomography Unveils Formation Mechanisms of Wear-Protective Tribofilms by ZDDP, Ionic Liquid, and Their Combination. ACS Appl. Mater. Interfaces.

[cit73] Qu J., Barnhill W. C., Luo H., Meyer 3rd H. M., Leonard D. N., Landauer A. K., Kheireddin B., Gao H., Papke B. L., Dai S. (2015). Synergistic effects between phosphonium-alkylphosphate ionic liquids and zinc dialkyldithiophosphate (ZDDP) as lubricant additives. Adv. Mater..

[cit74] Jiang W., Wang Y., Voth G. A. (2007). Molecular dynamics simulation of nanostructural organization in ionic liquid/water mixtures. J. Phys. Chem. B.

