# Supplementary material for: In situ nanoscale evaluation of pressure-induced changes in structural morphology of phosphonium phosphate ionic liquid at single-asperity contacts
Source: RSC Adv. 2021 Dec 22;12(1):413–9. doi: 10.1039/d1ra08026a (PMC8978665; doi:10.1039/d1ra08026a)
Supplement: RA-012-D1RA08026A-s001 [file RA-012-D1RA08026A-s001.pdf]

Electronic Supplementary Material (ESI) for RSC Advances.
This journal is © The Royal Society of Chemistry 2021

Supporting Information

In Situ Nanoscale Evaluation of Pressure-Induced Changes in Structural Morphology of Phosphonium Phosphate Ionic Liquid at Single-Asperity Contacts

Zixuan Li[1,2], Oscar Morales-Collazo[3], Robert Chrostowski[1,2], Joan F. Brennecke[3], Filippo Mangolini[1,4,]*

1. Texas Materials Institute, The University of Texas at Austin, Austin, TX 78712, USA
2. Materials Science and Engineering Program, The University of Texas at Austin, Austin, TX 78712, USA
3. McKetta Department of Chemical Engineering, The University of Texas at Austin, Austin, TX 78712, USA
4. Walker Department of Mechanical Engineering, The University of Texas at Austin, Austin, TX 78712, USA

* Author to whom correspondence should be addressed. Electronic email: filippo.mangolini@austin.utexas.edu

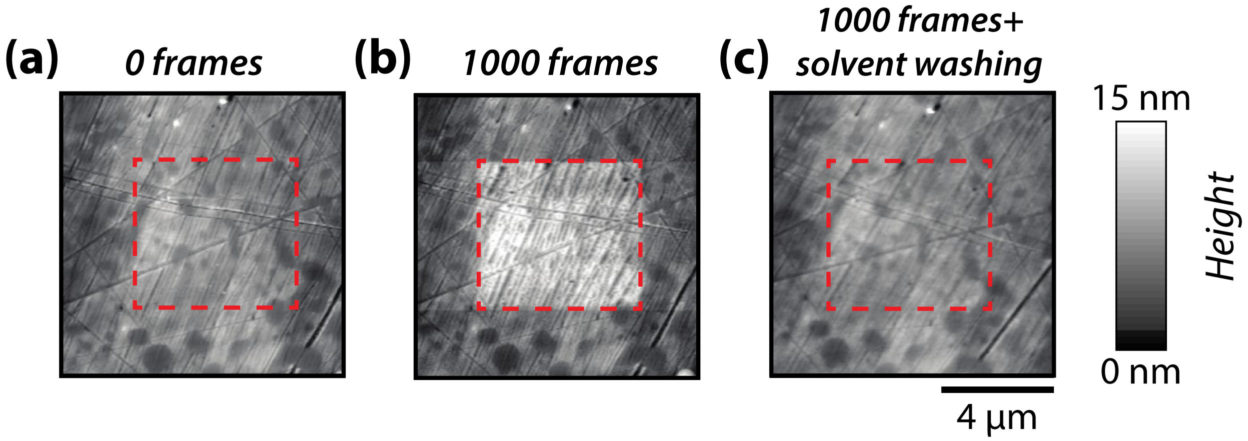

Figure S1. Topographic AFM images (10 x 10 μm^2) of an air-oxidized steel surface obtained using a DLC-coated silicon AFM tip. The topographic images were collected at a non-perturbative load. Height images (a) and (b) were acquired with the AFM tip immersed in trihexyltetradecylphosphonium bis(2-ethylhexyl) phosphate ([$P_{6,6,6,14}$][DEHP]) IL at 111 ± 1ºC. Additionally, image (b) was obtained after scanning the central part of the image with an applied pressure of 5.5 ± 0.3 GPa (highlighted with a red, dashed box) for 1000 frames. After the AFM experiments, the sample was washed with and sonicated in methanol and isopropanol to remove the supernatant [$P_{6,6,6,14}$][DEHP] IL. The height image (c) was collected in the same sample area of (a) and (b) and revealed the complete removal of the surface layer and the absence of any material removal from the underlying air-oxidized steel substrate.

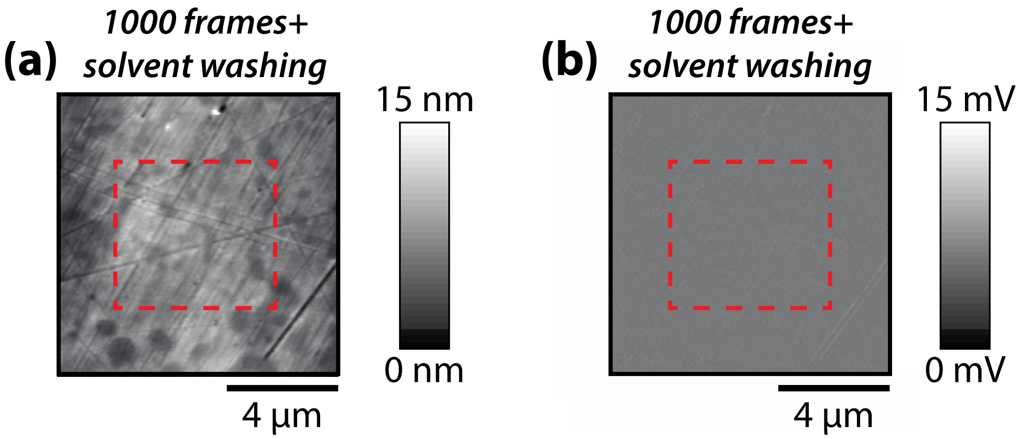

Figure S2. (a) Topographic AFM image (10 x 10 µm^2) of an air-oxidized steel surface obtained using a DLC-coated silicon AFM tip. The topographic image was collected at a non-perturbative load after scanning the central part of the image with an applied pressure of 5.5 ± 0.3 GPa (highlighted with a red, dashed box) for 1000 frames. After the AFM experiments, the sample was washed with and sonicated in methanol and isopropanol to remove the supernatant [$P_{6,6,6,14}$][DEHP] IL. The corresponding friction force map (b) was collected on the same sample. The absence of any contrast in the friction force map indicates that no chemical changes occurred in the area scanned with an applied pressure of 5.5 ± 0.3 GPa (highlighted with a red, dashed box) for 1000 frames.

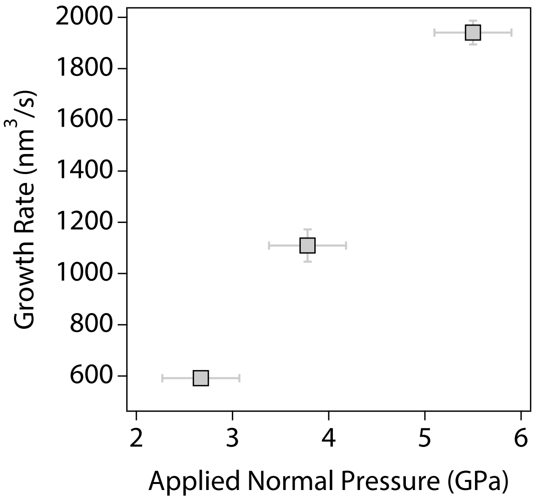

Figure S3. Dependence of the volumetric growth rate of the interfacial layer formed by [$P_{6,6,6,14}$][DEHP] on the normal applied pressure at 111 ± 1ºC. The growth rate was computed using the data displayed in Figure 3.

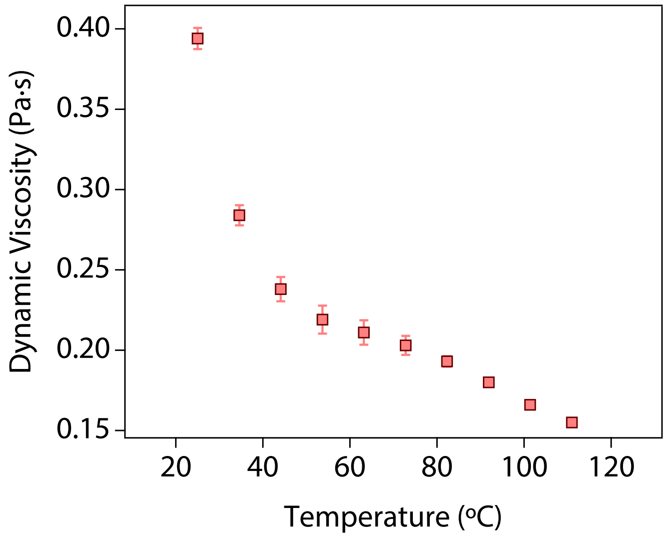

Figure S4. Dependence of the dynamic viscosity of [$P_{6,6,6,14}$][DEHP] on temperature. The viscosity measurement was conducted using a ATS NOVA Advanced Research Rheometer. The instrument was operated in stress control mode at a constant stress of 1 kPa with a 30 mm plate-plate configuration. Each measured temperature was allowed to equilibrate over 60 s. Integration time for each viscometry measurement was 75 s and a total of 4 measurements were taken for each temperature (points on the chart represent the average measured values, while error bars are the standard deviations).

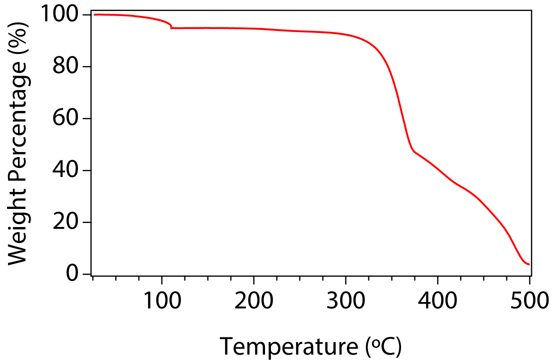

Figure S5. Thermogravimetric analysis (TGA) of [$P_{6,6,6,14}$][DEHP]. The sample was heated from room temperature to 110ºC with a rate of 20ºC, maintained at 110ºC for 45 min, and then heated up to 500ºC with a rate of 10ºC/min.
